# Intact Olfaction in a Mouse Model of Multiple System Atrophy

**DOI:** 10.1371/journal.pone.0064625

**Published:** 2013-05-17

**Authors:** Florian Krismer, Gregor K. Wenning, Yuntao Li, Werner Poewe, Nadia Stefanova

**Affiliations:** 1 Division of Neurobiology, Department of Neurology, Innsbruck Medical University, Innsbruck, Austria; 2 The Second School of Clinical Medicine, The Second Affiliated Hospital, Nanjing Medical University, Nanjing, China; Philadelphia VA Medical Center, United States of America

## Abstract

**Background:**

Increasing evidence suggests that olfaction is largely preserved in multiple system atrophy while most patients with Parkinson's disease are hyposmic. Consistent with these observations, recent experimental studies demonstrated olfactory deficits in transgenic Parkinson's disease mouse models, but corresponding data are lacking for MSA models.

**Methods:**

Olfactory function and underlying neuropathological changes were investigated in a transgenic multiple system atrophy mouse model based on targeted oligodendroglial overexpression of α-synuclein as well as wild-type controls. The study was divided into (1) a pilot study investigating olfactory preference testing and (2) a long-term study characterizing changes in the olfactory bulb of aging transgenic multiple system atrophy mice.

**Results:**

In our pilot behavioral study, we observed no significant differences in investigation time in the olfactory preference test comparing transgenic with wild-type animals. These findings were accompanied by unaffected tyrosine hydroxylase-positive cell numbers in the olfactory bulb. Similarly, although a significant age-related increase in the amount of α-synuclein within the olfactory bulb was detected in the long-term study, progressive degeneration of the olfactory bulb could not be verified.

**Conclusions:**

Our experimental data show preserved olfaction in a transgenic multiple system atrophy mouse model despite α-synucleinopathy in the olfactory bulb. These findings are in line with the human disorder supporting the concept of a primary oligodendrogliopathy with variable neuronal involvement.

## Introduction

Multiple system atrophy (MSA) is a rapidly progressive neurodegenerative disorder of unknown etiopathogenesis. It is characterized clinically by autonomic failure accompanied by parkinsonism and cerebellar ataxia [1]. The distinction of early stage MSA from related parkinsonian syndromes including Parkinson's disease (PD) can be challenging [2]. However, previous reports suggested that assessment of olfactory function is an important pointer in the differential diagnosis. MSA patients show intact or mildly impaired olfaction whereas most PD patients are hyposmic or sometimes anosmic [3]–[8]. Even more interestingly, olfactory disturbances may predate the onset of classic motor features in PD [9], [10]. Deficits in PD patients include impairment of odor detection, discrimination and identification [10], [11].

α-synuclein (αSYN) is a key protein in the pathogenesis of MSA and PD with the former being characterized by glial cytoplasmic inclusions (GCIs, Papp-Lantos bodies) and the latter by neuronal Lewy bodies as their subcellular hallmark feature. These αSYN-positive inclusions are also observed in the olfactory tract, predominantly affecting the anterior olfactory nucleus [12], [13]. In preclinical research, αSYN pathology may be replicated by transgenic (tg) ovexpression of αSYN under oligodendroglial [14]–[16] or neuronal promoters [17] mimicking MSA- or PD-like inclusion pathology, respectively.

Recently, olfactory disturbances have been studied in tg mouse models of PD. Behavioral alterations and olfactory bulb pathology in these models are reminiscent of the human disorder with age-related impairment in odor detection and discrimination [18]–[20] as well as extensive olfactory bulb pathology [21]–[23]. In contrast, smell disturbances in MSA models were only studied once in the context of glial derived neurotrophic factor (GDNF) replacement therapy [24]. This study reported olfactory impairment in tg versus wild-type (wt) animals in the saline-treated study arm; however, olfactory bulb pathology was not investigated [24].

In the present study, we investigated olfactory behavior and assessed neuropathological changes within the olfactory bulb (OB) and their age-related evolution in an established tg MSA mouse model featuring overexpression of αSYN in oligodendrocytes [14].

## Methods

The study was split into two parts: (1) a pilot study determining behavioral olfactory deficits and immunohistochemical differences in 9-months old animals and (2) a confirmatory long-term study (LTS) focusing on the analysis of OB aging. In the LTS, mice with 2, 6 and 18 months of age were studied. Both subprotocols compared homozygous tg MSA mice to age- and strain-matched non-littermate wt controls of the inbred C57BL/6 strain.

### Animals

The generation and characterization of tg mice with targeted overexpression of human αSYN (hαSYN) under the oligodendroglial proteolipid protein promotor (PLP-hαSYN) were described previously [14]. Tg and wt mice were originally obtained from P. Kahle (University of Tübingen, Tübingen, Germany) and Charles River Laboratories (Charles River Laboratories, Sulzfeld, Germany), respectively. Mice were bred and maintained in a temperature-controlled specific pathogen free room with a 12-h light/dark cycle and free access to food and water at the Animal Facility of Innsbruck Medical University. Genotyping was performed by tail clip polymerase chain reaction (PCR) using the following primers: Forward: 5′-ATG GAT GTA TTC ATG AAA GG-3′; reverse: 5′-TTA GGC TTC AGG TTC GTA G-3′.

This study was carried out in strict accordance with the Austrian guidelines for the care and use of laboratory animals and all in vivo protocols were approved by the Austrian Federal Ministry of Science and Research (do. Zi. 6001). All efforts were made to minimize the number of animals used and their suffering.

### Behavioral testing

We performed olfactory preference testing in 9 month old mice according to a previously published protocol [25], [26]. This test is designed to identify specific odor detection deficiencies, based on the inability to sense attractive scents [25], [26]. Briefly, four home cages sized 26 cm×45 cm×20 cm (width × length × height) were lined up next to each other separated by opaque filter paper. Animals were habituated to the unknown surrounding of an empty cage for a period of 1 hour with transfer steps to the next cage every 15 minutes. Moreover, a video camera was placed such that the entire arena cage ( =  last habituation cage) was in focus. Thereafter, 5 cm×5 cm squares of scented filter paper were placed on the opposite end of the mice's current position. The animal's behavior was recorded on video for a period of 3 minutes. Different scents were randomly presented at an interval of at least one minute. The following odorants were used: distilled water (control, inherent smell of the filter paper), peanut butter (Skippy, Unilever) dissolved in mineral oil (Sigma-Aldrich, Vienna, Austria), vanilla (Oetker GmbH, Bielefeld, Germany) and cinnamon (Invero, Wiener Neudorf, Austria) dissolved in mineral oil. Test cages were extensively cleaned after each mouse. Finally, to avoid confounding of data owing to task learning, mice performed the test once only.

### Tissue preparation and immunohistochemistry

Mice were sacrificed at the designated time points by transcardial perfusion with 10 ml of 0.1 M phosphate buffered saline (PBS; Sigma-Aldrich, Vienna, Austria) followed by ice-cold 4% paraformaldehyde (PFA; Merck, Darmstadt, Germany) in PBS under deep thiopental (Sandoz, Kundl, Austria) anesthesia (i.e. 120 mg/kg body-weight thiopental). Brains were quickly removed and post-fixed in 4% PFA dissolved in PBS at 4°C over night. After cryoprotection in 25% PBS-sucrose solution (Sigma-Aldrich, Vienna, Austria), brains were slowly frozen in 2-methylbutane (Merck, Darmstadt, Germany) and stored at -80°C until further processing.

Serial coronal sections (40 µm) were cut on a freezing microtome (Leica, Nussloch, Germany). One series of sections per animal was mounted on slides and underwent cresyl violet (CV) staining. Free floating sections were stained according to a standard immunoperoxidase protocol [27], [28]. The following primary antibodies were used: mouse anti-tyrosine hydroxylase (TH, 1∶1000; Sigma, St. Louis, Missouri, USA), rat anti-mouse CD11b (1∶150; Serotec, Oxford, UK), rat anti-human-α-synuclein (15G7, 1∶200; Enzo Life Sciences, Exeter, UK). For immunohistochemistry, secondary antibodies were biotinylated anti-mouse or anti-rat IgG (Vector Laboratories, Burlingame, California, USA) as appropriate. Following incubation with avidin-biotin complex (ABC) reagent (Vectastain ABC kit, Vector Laboratories, Burlingame, California, USA), immunohistochemical reactions were visualized by 3,3′-diamino-benzidine-tetrahydrochloride (DAB; Sigma, St. Louis, Missouri, USA). For immunofluorescence, Alexa 488- or Alexa 594-conjugated anti-rat or anti-mouse IgG (Molecular Probes, Life Technologies, Paisley, UK), as appropriate, were applied as secondary antibodies.

### Image analysis

Image analyses were performed by a blinded investigator. Nikon E-800 (Nikon, Vienna, Austria) microscope equipped with a digital camera (Nikon DXM 1200, Nikon, Vienna, Austria) connected to a computer-assisted analysis system (Stereo Investigator Software, MicroBrightField Europe, Magdeburg, Germany) was used. Regions of interest were outlined manually according to the Paxinos and Franklin Mouse Brain Atlas (1997, Academic Press, San Diego). The optical fractionator workflow was exploited to generate an unbiased estimate of TH- and 15G7-immunoreactive cell numbers in the granular layer and glomerular layer of the OB. Microglial activation was determined by measuring optical densitiy (OD) of CD11b immunoreactivity. Briefly, staining brightness was measured in the glomerular and granular layer of the OB (OD_ROI_) and a blank area (OD_Background_). Next, the OD ratio was calculated according to the following formula: OD ratio  =  −log (OD_ROI_/OD_Background_) as previously described.[27] OB atrophy was evaluated by outlining the OB bilaterally on 3 adjacent CV stained sections and measuring the respective area using Stereo Investigator Software.

For immunofluorescence, imaging was performed using a DMI 4000B Leica microscope equipped with Digital Fire Wire Color Camera DFC300 FX and Application Suite V3.1 software by Leica (Leica, Nussloch, Germany).

### Statistical analysis

Statistical analysis was performed using SPSS 20.0 (SPSS Inc., Chicago, Illinois, USA). If not stated otherwise, data are expressed as mean ± standard error of mean (SEM). Group differences were analyzed by Student's T-test, Kruskal-Wallis-Test, one-way analysis of variance (ANOVA) or two-way ANOVA and Bonferroni correction of multiple comparisons as appropriate. The significance level was set at p<0.05; all tests were two-sided.

## Results

### Pilot study: olfactory preference test in aged MSA mice

**Figure 1:**
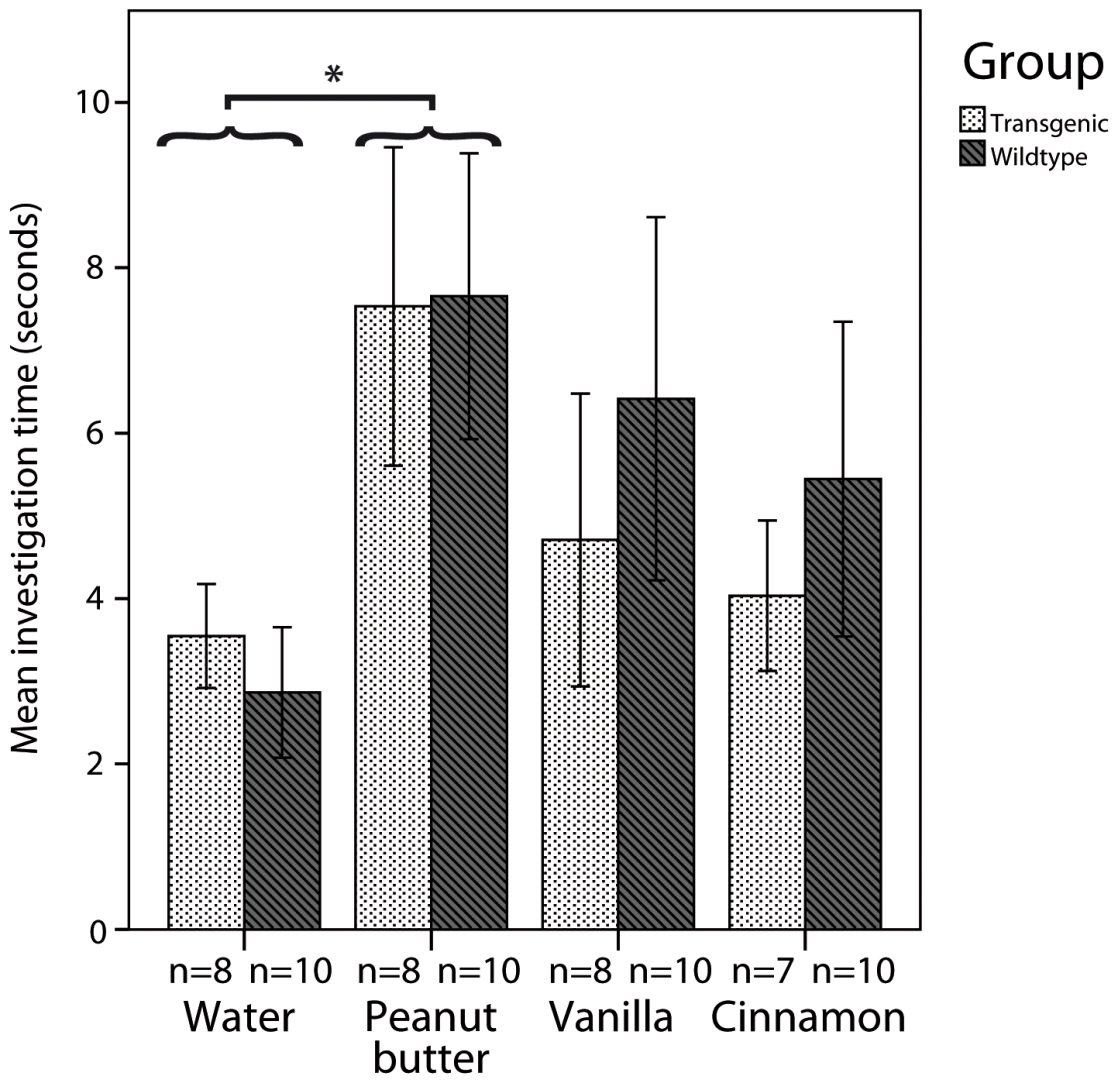
Olfactory behavior. Mean investigation time in seconds in olfactory preference testing of 9 months old mice. Data are expressed as mean; error bars indicate the standard error of mean. Sample sizes are reported below the X-axis. * P<0.05.

In the olfactory preference testing paradigm, there was no significant difference in investigation time between 9-month old tg and wt animals (F_1, 16_ = 0.000, p = 0.989, ANOVA). Although the different scents were a non-significant term in our ANOVA model (F_3, 48_ = 2.189, p = 0.101, ANOVA), pairwise comparisons revealed that the inherent smell of the filter paper (water sample) was less attractive than peanut butter to the animals irrespective of the underlying genotype (p = 0.041, post-hoc analysis of ANOVA model with Bonferroni correction for multiple comparisons) (Figure 1).

Immunohistochemical analysis of TH Immunoreactive cells within the glomerular layer of the OB yielded no difference in the number of dopaminergic neurons in 9 months old mice (p = 0.129, Student's T-test, Table 1). In addition, stereological analysis of 15G7-immunoreactive cells revealed αSYN pathology of the OB in 9 months old mice already (Table 1).

**Table 1:** Stereological cell counts of 15G7 and TH immunohistochemistry.

Age	Genotype	15G7, mean ± SEM (n)	TH, mean ± SEM (n)	p-value^1^
*2 months*	Transgenic	77532.1±7258.3 (n = 5)	67893.9±8327.7 (n = 5)	0.226
*2 months*	Wildtype	Not applicable	53583.1±5948.7 (n = 4)	
*6 months*	Transgenic	108711.4±11531.2 (n = 6)	73244,8±10118.7 (n = 6)	0.619
*6 months*	Wildtype	Not applicable	82077.8±13940.0 (n = 6)	
*9 months*	Transgenic	160520.2±10012.3 (n = 5)^2,3^	69613,5±17201.7 (n = 6)	0.129
*9 months*	Wildtype	Not applicable	111041.0±18096.1 (n = 7)	
*18 months*	Transgenic	124320.8±10888.0 (n = 8)^2^	75983.0±7448.5 (n = 8)	0.197
*18 months*	Wildtype	Not applicable	62436.0±5622.8 (n = 6)	

π… Data from the pilot study;

1 … Comparing TG vs. WT animals in TH IHC by Student's T Test;

2 … p<0.05 compared to 2 month old mice (Mann-Whitney U Test with Bonferroni correction for multiple comparisons);

3 … p<0.05 compared to 6 month old mice (Mann-Whitney U Test with Bonferroni correction for multiple comparisons). 15G7… human αSYN, TH… tyrosine hydroxylase, TG… transgenic, WT… wildtype, IHC… immunohistochemistry, SEM… standard error of mean.

### Long-term study: OB ageing

As indicated above, the LTS involved assessment of neuropathology in 2, 6 and 18 month-old animals to detect age- and αSYN-related neurodegeneration in the OB.

**Figure 2:**
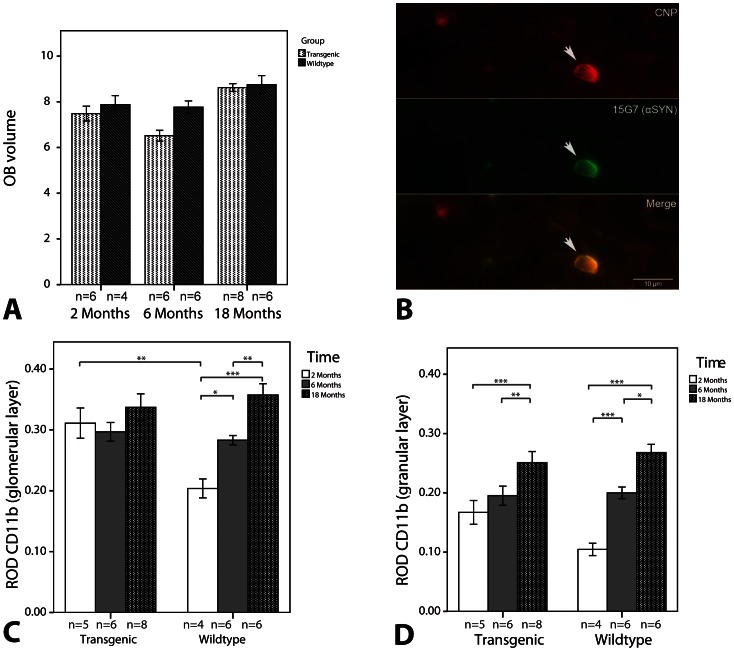
Immunohistochemistry of confirmatory long-term study. A…OB volume; B… Immunofluorescence for CNPase (red) and 15G7 (green) confirmed αSYN expression in glial cells (arrow), scale bar: 10 µm; ROD of CD11b immunohistochemistry in the (C) glomerular layer and the (D) granular layer of the OB; All data are expressed as mean; error bars indicate the standard error of mean. Sample sizes are reported below the X-axis; * P<0.05, ** P<0.01, *** P<0.001.

We observed an age-dependent accumulation of hαSYN immunoreactive inclusions in the OB of tg mice (p = 0.036, Kruskal-Wallis; Table 1). However, the highest number of MSA-like cytoplasmic inclusion patholgoy was observed in 9 months old animals (Table 1) with a statistically significant difference to 2 months (p = 0.048, Mann-Whitney-U-Test with Bonferroni correction for multiple comparisons) and 6 months (p = 0.024, Mann-Whitney-U-Test with Bonferroni correction for multiple comparisons) old animals, but not compared to 18 months old mice (p = 0.558, Mann-Whitney-U-Test with Bonferroni correction for multiple comparisons). Transgenic hαSYN driven by the PLP promoter was detected in CNPase-positive glial cells of the OB (Figure 2b) similar to other CNS regions [29], [30]. The increase in αSYN load did not convert into OB degeneration, in particular, there were no significant differences in OB volume between the two genotypes (F_1, 39_ = 3.263, p = 0.079, ANOVA; Figure 2a). Likewise, the number of TH-ir cells remained stable over time (F_3, 39_ = 1.248, p = 0.305, ANOVA; Table 1) and genotype was a non-significant term in the ANOVA model (F_1, 39_ = 0.377, p = 0.543, ANOVA; Table 1).

In contrast, tg animals featured early OB microglial activation with significantly increased CD11b immunoreactivity in the glomerular layer at 2 months compared to wt animals (p<0.01, ANOVA model with Bonferroni correction). However, wt animals showed an age-related increase in microglial activation catching up the difference which was present at 2 months (Figure 2c). In the granular layer, we observed age-related enhancement of CD11b immunoreactivity in tg as well as wt animals (F_3, 38_ = 21.5, p<0.001, ANOVA) with differences between the two genotypes failing to reach statistical significance (Figure 2d).

## Discussion

The presence of olfactory deficits is an important diagnostic pointer in patients presenting with parkinsonism. MSA patients show largely preserved olfactory function whereas most PD patients are hyposmic [3]–[8]. In preclinical research, olfaction has been extensively studied in PD αSYN mouse models revealing deficits of odor detection and discrimination [18]–[20]. In addition, extensive olfactory bulb pathology has been reported in PD αSYN mouse models [21]–[23].

In the present study, we explored olfactory function and OB pathology in a transgenic mouse model with targeted oligodendroglial overexpression of hαSYN driven by the PLP promoter [14] featuring MSA-like inclusion pathology. Our data clearly show that olfactory preference testing, a widely used behavioral paradigm to identify specific olfactory deficiencies (i.e. the ability to sense attractive scents) [25], [26], was not impaired in the PLP-hαSYN mouse model. This finding is in contrast to a previous study in a related mouse model overexpressing αSYN under the myelin basic protein (MBP) promoter which reported increased pellet retrieval latencies in tg compared to wt animals [24]. The discrepancy might be due to differences between the models used - including myelination with PLP-hαSYN mice lacking obvious demyelination at ages up to 18 months [14] and MBP-hαSYN mice showing myelin damage at young ages already [15]. Other methodological issues (i.e. olfactory preference testing versus buried pellet test) might also have contributed to the observed differences. To verify that the absence of obvious smell deficits in PLP-hαSYN mice is a reliable observation, we performed neuropathological work-up demonstrating lack of genotype-specific OB atrophy as well as lack of accelerated neuronal loss in the transgenic OB, despite a trend towards a lower TH-immunoreactive cell number within the OB of 9 months old tg animals. The PLP-hαSYN transgenic model reproduces MSA-like selective vulnerability of the different subpopulations of dopaminergic neurons in SNc and OB. Putting these considerations into a clinical perspective, it has to be emphasized that previous clinical studies in human MSA found varying degrees of olfactory deficits with impairment being less pronounced in MSA compared to PD [3], [7], [31]–[33]. In addition, αSYN inclusion pathology has been demonstrated in the human OB [12], however, a recent clinicopathological case series could not identify any smell deficit in MSA [5].

To exclude oversights due to age-related neurodegeneration, we subsequently conducted a long-term confirmatory study focusing on neuropathological read-outs. Age-dependent accumulation of hαSYN immunoreactive inclusions with a significant increase at 9 and 18 months compared 2 months was observed in transgenic OB. This finding is partly in agreement with the human disorder showing GCIs in the OB [12], however, the temporal evolution of GCI pathology in the OB has not been studied in MSA patients so far. Surprisingly, the inclusion pathology did not convert into neurodegeneration. Neither olfactory bulb volume nor TH-ir cell numbers within the glomerular layer of the OB were significantly different between wt and tg animals. In the glomerular layer of the OB, microglial activation was more pronounced in tg compared to wt animals at 2 months of age. However, there was no age-related effect on CD11b immunoreactivity in tg animals, whereas microglial activation continuously increased in wt animals. In the granular layer, age-related enhancement of CD11b immunoreactivity was observed in both, tg and wt animals. These findings are in line with a previous study reporting early and sustained microglial activation affecting the striatum and the SNc of PLP-hαSYN mice [34]. Finally, it has to be acknowledged that additional olfactory tests may be helpful to study independent functional domains associated with olfaction in 9 months old mice and further longitudinal studies are required to exclude late-onset olfactory deficits paralleling the progressive OB α-synucleinopathy that has been observed in the present study. However, preserved olfaction at 9 months of age clearly separates the PLP-hαSYN MSA mouse model from corresponding PD mouse models [18], [19]. Semi-quantitative analysis of microglial activation by OD measurements of CD11b immunostainings may be affected by various factors; therefore, we applied counter-measures including (1) the calculation of relative OD values to account for different labeling intensities, and (2) the acquisition of all images during a single microscopy session at uniform microscopy settings to vigorously control confounding factors.

To the best of our knowledge this is the first analysis of olfactory behavior as well as candidate neuropathology in the context of a transgenic MSA mouse model. Our experimental data suggest preserved olfactory function providing further support to a recent notion claiming that olfactory deficits are unlikely in MSA [5] reflecting the unique oligodendrogliopathy.

## References

[1] Wenning GK, Colosimo C, Geser F, Poewe W (2004) Multiple system atrophy. Lancet Neurol 3: 93–103. 14747001 10.1016/s1474-4422(03)00662-8

[2] Hughes AJ, Daniel SE, Ben-Shlomo Y, Lees AJ (2002) The accuracy of diagnosis of parkinsonian syndromes in a specialist movement disorder service. Brain 125: 861–870. 11912118 10.1093/brain/awf080

[3] Wenning GK, Shephard B, Hawkes C, Petruckevitch A, Lees A, et al (1995) Olfactory function in atypical parkinsonian syndromes. Acta Neurol Scand 91: 247–250. 7625148 10.1111/j.1600-0404.1995.tb06998.x

[4] Suzuki M, Hashimoto M, Yoshioka M, Murakami M, Kawasaki K, et al (2011) The odor stick identification test for Japanese differentiates Parkinson's disease from multiple system atrophy and progressive supra nuclear palsy. BMC Neurol 11: 157. 22192419 10.1186/1471-2377-11-157 PMC3297535

[5] Glass PG, Lees AJ, Mathias C, Mason L, Best C, et al (2012) Olfaction in pathologically proven patients with multiple system atrophy. Mov Disord 27: 327–328. 21953531 10.1002/mds.23972

[6] Kikuchi A, Baba T, Hasegawa T, Sugeno N, Konno M, et al (2011) Differentiating Parkinson's disease from multiple system atrophy by [123I] meta-iodobenzylguanidine myocardial scintigraphy and olfactory test. Parkinsonism Relat Disord 17: 698–700. 21840242 10.1016/j.parkreldis.2011.07.011

[7] Garland EM, Raj SR, Peltier AC, Robertson D, Biaggioni I (2011) A cross-sectional study contrasting olfactory function in autonomic disorders. Neurology 76: 456–460. 21282592 10.1212/WNL.0b013e31820a0caf PMC3034411

[8] Doty RL (2012) Olfactory dysfunction in Parkinson disease. Nat Rev Neurol 8: 329–339. 22584158 10.1038/nrneurol.2012.80

[9] Ross GW, Petrovitch H, Abbott RD, Tanner CM, Popper J, et al (2008) Association of olfactory dysfunction with risk for future Parkinson's disease. Ann Neurol 63: 167–173. 18067173 10.1002/ana.21291

[10] Doty RL, Stern MB, Pfeiffer C, Gollomp SM, Hurtig HI (1992) Bilateral olfactory dysfunction in early stage treated and untreated idiopathic Parkinson's disease. J Neurol Neurosurg Psychiatry 55: 138–142. 1538221 10.1136/jnnp.55.2.138 PMC488979

[11] Doty RL, Deems DA, Stellar S (1988) Olfactory dysfunction in parkinsonism: a general deficit unrelated to neurologic signs, disease stage, or disease duration. Neurology 38: 1237–1244. 3399075 10.1212/wnl.38.8.1237

[12] Kovacs T, Papp MI, Cairns NJ, Khan MN, Lantos PL (2003) Olfactory bulb in multiple system atrophy. Mov Disord 18: 938–942. 12889086 10.1002/mds.10466

[13] Pearce RK, Hawkes CH, Daniel SE (1995) The anterior olfactory nucleus in Parkinson's disease. Mov Disord 10: 283–287. 7651444 10.1002/mds.870100309

[14] Kahle PJ, Neumann M, Ozmen L, Muller V, Jacobsen H, et al (2002) Hyperphosphorylation and insolubility of alpha-synuclein in transgenic mouse oligodendrocytes. EMBO Rep 3: 583–588. 12034752 10.1093/embo-reports/kvf109 PMC1084143

[15] Shults CW, Rockenstein E, Crews L, Adame A, Mante M, et al (2005) Neurological and neurodegenerative alterations in a transgenic mouse model expressing human alpha-synuclein under oligodendrocyte promoter: implications for multiple system atrophy. J Neurosci 25: 10689–10699. 16291942 10.1523/JNEUROSCI.3527-05.2005 PMC6725840

[16] Yazawa I, Giasson BI, Sasaki R, Zhang B, Joyce S, et al (2005) Mouse model of multiple system atrophy alpha-synuclein expression in oligodendrocytes causes glial and neuronal degeneration. Neuron 45: 847–859. 15797547 10.1016/j.neuron.2005.01.032

[17] Rockenstein E, Mallory M, Hashimoto M, Song D, Shults CW, et al (2002) Differential neuropathological alterations in transgenic mice expressing alpha-synuclein from the platelet-derived growth factor and Thy-1 promoters. J Neurosci Res 68: 568–578. 12111846 10.1002/jnr.10231

[18] Kim YH, Lussier S, Rane A, Choi SW, Andersen JK (2011) Inducible dopaminergic glutathione depletion in an alpha-synuclein transgenic mouse model results in age-related olfactory dysfunction. Neuroscience 172: 379–386. 21055449 10.1016/j.neuroscience.2010.10.072 PMC3010458

[19] Fleming SM, Tetreault NA, Mulligan CK, Hutson CB, Masliah E, et al (2008) Olfactory deficits in mice overexpressing human wildtype alpha-synuclein. Eur J Neurosci 28: 247–256. 18702696 10.1111/j.1460-9568.2008.06346.x PMC3108548

[20] Taylor TN, Caudle WM, Shepherd KR, Noorian A, Jackson CR, et al (2009) Nonmotor symptoms of Parkinson's disease revealed in an animal model with reduced monoamine storage capacity. J Neurosci 29: 8103–8113. 19553450 10.1523/JNEUROSCI.1495-09.2009 PMC2813143

[21] Ubeda-Banon I, Saiz-Sanchez D, de la Rosa-Prieto C, Mohedano-Moriano A, Fradejas N, et al (2010) Staging of alpha-synuclein in the olfactory bulb in a model of Parkinson's disease: cell types involved. Mov Disord 25: 1701–1707. 20574962 10.1002/mds.23197

[22] Nuber S, Petrasch-Parwez E, Arias-Carrion O, Koch L, Kohl Z, et al (2011) Olfactory neuron-specific expression of A30P alpha-synuclein exacerbates dopamine deficiency and hyperactivity in a novel conditional model of early Parkinson's disease stages. Neurobiol Dis 44: 192–204. 21767644 10.1016/j.nbd.2011.06.017

[23] Tofaris GK, Garcia Reitbock P, Humby T, Lambourne SL, O'Connell M, et al (2006) Pathological changes in dopaminergic nerve cells of the substantia nigra and olfactory bulb in mice transgenic for truncated human alpha-synuclein(1-120): implications for Lewy body disorders. J Neurosci 26: 3942–3950. 16611810 10.1523/JNEUROSCI.4965-05.2006 PMC6673887

[24] Ubhi K, Rockenstein E, Mante M, Inglis C, Adame A, et al (2010) Neurodegeneration in a transgenic mouse model of multiple system atrophy is associated with altered expression of oligodendroglial-derived neurotrophic factors. J Neurosci 30: 6236–6246. 20445049 10.1523/JNEUROSCI.0567-10.2010 PMC2896284

[25] Witt RM, Galligan MM, Despinoy JR, Segal R (2009) Olfactory behavioral testing in the adult mouse. J Vis Exp 23: 949. 10.3791/949 PMC2782999 19229182

[26] Kobayakawa K, Kobayakawa R, Matsumoto H, Oka Y, Imai T, et al (2007) Innate versus learned odour processing in the mouse olfactory bulb. Nature 450: 503–508. 17989651 10.1038/nature06281

[27] Stefanova N, Kaufmann WA, Humpel C, Poewe W, Wenning GK (2012) Systemic proteasome inhibition triggers neurodegeneration in a transgenic mouse model expressing human alpha-synuclein under oligodendrocyte promoter: implications for multiple system atrophy. Acta Neuropathol 124: 51–65. 22491959 10.1007/s00401-012-0977-5 PMC3377902

[28] Stefanova N, Georgievska B, Eriksson H, Poewe W, Wenning GK (2012) Myeloperoxidase inhibition ameliorates multiple system atrophy-like degeneration in a transgenic mouse model. Neurotox Res 21: 393–404. 22161470 10.1007/s12640-011-9294-3

[29] Stefanova N, Hainzer M, Stemberger S, Couillard-Despres S, Aigner L, et al (2009) Striatal transplantation for multiple system atrophy–are grafts affected by alpha-synucleinopathy? Exp Neurol 219: 368–371. 19460374 10.1016/j.expneurol.2009.05.016

[30] Stemberger S, Poewe W, Wenning GK, Stefanova N (2010) Targeted overexpression of human alpha-synuclein in oligodendroglia induces lesions linked to MSA-like progressive autonomic failure. Exp Neurol 224: 459–464. 20493840 10.1016/j.expneurol.2010.05.008 PMC2913120

[31] Nee LE, Scott J, Polinsky RJ (1993) Olfactory dysfunction in the Shy-Drager syndrome. Clin Auton Res 3: 281–282. 8292885 10.1007/BF01829019

[32] Muller A, Mungersdorf M, Reichmann H, Strehle G, Hummel T (2002) Olfactory function in Parkinsonian syndromes. J Clin Neurosci 9: 521–524. 12383407 10.1054/jocn.2001.1071

[33] Goldstein DS, Sewell L (2009) Olfactory dysfunction in pure autonomic failure: Implications for the pathogenesis of Lewy body diseases. Parkinsonism Relat Disord 15: 516–520. 19201246 10.1016/j.parkreldis.2008.12.009 PMC4164391

[34] Stefanova N, Reindl M, Neumann M, Kahle PJ, Poewe W, et al (2007) Microglial activation mediates neurodegeneration related to oligodendroglial alpha-synucleinopathy: implications for multiple system atrophy. Mov Disord 22: 2196–2203. 17853477 10.1002/mds.21671

